# Holocene pollen and phytolith dataset from the Xinglong site in the Bashang Grassland, northern China

**DOI:** 10.1016/j.dib.2025.112350

**Published:** 2025-12-03

**Authors:** Guanyu Wang, Zhenwei Qiu, Lina Zhuang, Huiyun Rao, Zhihua Yang, Wenhui Liu

**Affiliations:** a National Museum of China, Beijing, 100006, China; b Key Laboratory of Vertebrate Evolution and Human Origins, Institute of Vertebrate Paleontology and Paleoanthropology, Chinese Academy of Sciences, Beijing, 100044,China

**Keywords:** Pollen, Phytolith, Bashang Grassland, Early-mid holocene, Regional vegetation;Environmental history

## Abstract

This dataset comprises pollen and phytolith records obtained from the Xinglong site (42.09°N, 114.61°E) in the Bashang Grassland, northern China. The data were systematically collected from a 340 cm sediment profile (TG1E) containing well-stratified deposits from the Paleolithic-Neolithic transition to mid-Neolithic period (13,500–5000 cal. a BP). 70 contiguous samples were taken in 5 cm interval for multi-proxy analyses. The dataset provides fundamental material for reconstructing early-mid Holocene environment and human-environment interactions.

Pollen extraction followed standardized HCl-NaOH-HF protocols using *Lycopodium* spore tracers, with subsequent sieving, centrifuging and preserving in glycerol. Microscopic analysis identified 29,447 pollen and spore grains (average approximately 421 grains/sample), including 27,729 grains of angiosperm pollen, 1622 grains of fern spores, 76 grains of algae, 20 grains of bryophyte spores, representing 66 plant taxa, including woody species (16 taxa), herbaceous plants (41 taxa), fern types (7 taxa), algae (1 taxon) and bryophyte(1 taxon).

Phytolith extraction employed H_2_O_2_-HCl-ZnBr_2_ protocols with *Lycopodium* tracers, followed by sieving, centrifuging, ethanol dehydration and mounting in Canada balsam. Microscopic analysis revealed 21,344 specimens (average about 305 grains/sample), dominated by bulliform, square, rectangular, elongate psilate, bilobate, acicular hair cell and rondel from species such as *Phragmites australis*, Eragrostoideae, Panicoideae, and Pooideae.

All raw data are provided in .xls format.

This dataset offers significant reuse potential for: (1) Investigating early rainfed agricultural development patterns in northern China; (2) Analyzing regional vegetation and environmental history; (3) Modeling human occupation dynamics and human adaptation strategies during the Paleolithic-Neolithic transition; (4) Conducting comparative studies with other paleoenvironmental records across East Asia;(5) Serving as a methodological reference for future archaeobotanical research in similar regions.

Specifications Table

**Table:** 

Subject	Earth & Environmental Sciences
Specific subject area	Quaternary environment, Environmental archaeology, Archaeobotany
Type of data	Table(.xls format) Raw.
Data collection	Pollen data were identified and counted≥300 grains per sample at 400 × using Olympus BX53 optical microscope. Phytolith data were identified and counted ≥300 grains per sample at 400 × under a Nikon Eclipse LV100N POL optical microscope.
Data source location	The data were collected from the Xinglong site (42.09° N, 114.61°E), Zhaoyanghe Town, Kangbao County, Hebei Province, China
Data accessibility	Repository name: Holocene pollen and phytolith data from the Xinglong site in the Bashang Grassland, northern China Data identification number: 10.17632/5k3294k329.1 Direct URL to data: https://data.mendeley.com/datasets/5k3294k329/1
Related research article	Z. Qiu, L. Zhuang, H. Rao, Z. Yang, W. Liu, G. Wang, 2025. Ecological environment of early-mid Holocene millet cultivation in northern China: Insights from the Xinglong site. Quaternary Sci. Rev. 356, 109,295. https://doi.org/10.1016/j.quascirev.2025.109295

## 1 Value of the Data

• Why are these data valuable?
  ○ Pollen and phytolith not only play a significant role in reconstructing ancient vegetation landscape but also serve as crucial indicators for revealing climate and environmental changes. The Xinglong site is located in the Bashang region, situated in a transitional zone between the Inner Mongolian Plateau, the Yanshan Mountains, and the North China Plain. It is also a transitional zone between monsoon climate and continental climate, arid and semi-arid region, temperate hardwood forest and grassland, agricultural area and pastoral area, which is shaped by the combined effects of latitude, altitude, and monsoon. Research on pollen and phytolith from the Xinglong site can contribute to elucidating and interpreting the climatic conditions and vegetation succession of the site, understanding regional vegetation and environmental history during the early to mid-Holocene in the Bashang Grassland in the Inner Mongolia Plateau, northern China, thus providing solid data support for the related research.
• How can these data be reused by other researchers?
  ○ Archaeological pollen and phytolith records effectively serve to reconstruct local paleovegetation and paleoenvironment characteristics. These data are useful in elucidating the spatiotemporal patterns of early rainfed agriculture, the climatic and environmental shifts, and human-environment interactions during the Paleolithic-Neolithic transition up to the mid-Neolithic period.
• These data offer significant reuse potential in the following ways:
  ○ Early agricultural aspect: Help investigate the origin and early development of rainfed millet agriculture in northern China;
  ○ Regional vegetation and environmental history aspect: Help reconstruct spatiotemporal patterns of vegetation and climate variability in Bashang Grassland;
  ○ Human-environment interaction aspect: Offer evidence for understanding how early human adapted to environmental shifts during the Paleolithic-Neolithic transition and contribute to debates on human subsistence strategies;
  ○ Comparative studies aspect: Enable cross regional comparisons with other archaeological and paleoclimatic datasets in East Asia;
  ○ Methodological reference aspect: Document standardized protocols for pollen and phytolith extraction, which is useful for future archaeobotanical research, and provide reference for modern ecological protection, climate change response and sustainable agricultural development.

## 2 Background

This dataset was compiled to fill in key gaps in paleoenvironmental records from the Bashang Grassland transitional region. The primary motivation was to provide a robust, multi-proxy evidence combining phytolith and pollen data for examining Holocene environmental variability in northern China. These data were generated by the need for high-resolution records of the Bashang region during the Paleolithic-Neolithic transition and the absence of systematic phytolith-pollen datasets in the relevant zone.

The dataset contributes the associated research article by providing full stratigraphic context for all samples, detailed methodological protocols for dual-proxy analysis, high-resolution phytolith and pollen raw data and comprehensive taxonomic identification records. This helps to establish three pivotal phases in the Holocene climatic evolution of the Xinglong region in the original research article.

## 3 Data Description

The files associated with this Data-in-Brief article includes:

(1) The raw pollen data in .xls format (Holocene pollen data from the Xinglong site in the Bashang Grassland, northern China.xls): A total of 29,447 grains of spore and pollen were identified and counted in the profile, with a total concentration of approximately 51,457 grains/g, including 27,729 grains of angiosperm pollen, 1622 grains of fern spores, 76 grains of algae, 20 grains of bryophyte spores, and an average of approximately 421 grains per sample. In general, sixty-six taxa were identified, including sixteen woody species, forty one herbaceous plants, seven fern types, one algae, and one bryophyte. Arboreal taxa mainly included *Pinus, Picea, Betula, Carpinus, Quercus, Ulmus, Tilia, Acer, Tamarix, Hippophae, Nitraria, Ilex, Spiraea, Ephedra*, Pyrolaceae and Elaeagnaceae. Terrestrial herbaceous vegetation featured Poaceae, Chenopodiaceae, *Artemisia, Chrysanthemum, Saussurea*, Asteraceae, *Taraxacum, Aster*, Urticaceae, Euphorbiaceae, Brassicaceae, Scrophulariaceae, Campanulaceae, Rosaceae, *Sanguisorba, Potentilla*, Leguminosae, *Polygonum*, Labiatae, *Thalictrum, Ranunculus*, Ranunculaceae, *Macleaya*, Valerianaceae, Umbelliferae, Papaveracea, Convolvulaceae, *Galium, Geranium*, Liliaceae, Solanaceae, *Saxifraga, Xanthium*, Rubiaceae, Asclepiadaceae, Crassulaceae, Plumbaginaceae, Caryophyllaceae, *Humulus* and Cyperaceae. Aquatic herbaceous plant is *Typha*. Ferns mainly included *Selaginella Sinensis, Selaginella, Adiantum*, Polypodiaceae, *Lycopodium*, Monolete spores and Leiotriletes. Algae is *Zygnema* and bryophyte is Ricciaceae.

(2) The raw phytolith data in .xls format (Holocene phytolith data from the Xinglong site in the Bashang Grassland, northern China): Seventy phytolith samples (6–340 cm depth) contained 21,344 counted specimens, averaging about 305 grains per sample, and the total concentration is 36,146 grains/g per sample (ranging from 3149 to 124,330 grains/g). Dominant morphotypes include bulliform, square, rectangular, short-saddle, bilobate, elongate psilate, elongate echinate, elongate dendritic, acicular hair cell, rondel, trapeziform sinuate, and polyhedron aggregate. There are also a small number of sponge spicules. The identified plant species mainly include *Phragmites australis*, Eragrostoideae, Panicoideae, and Pooideae.

## 4 Experimental Design, Materials and Methods

### 4.1 Location

**Fig. 1:**
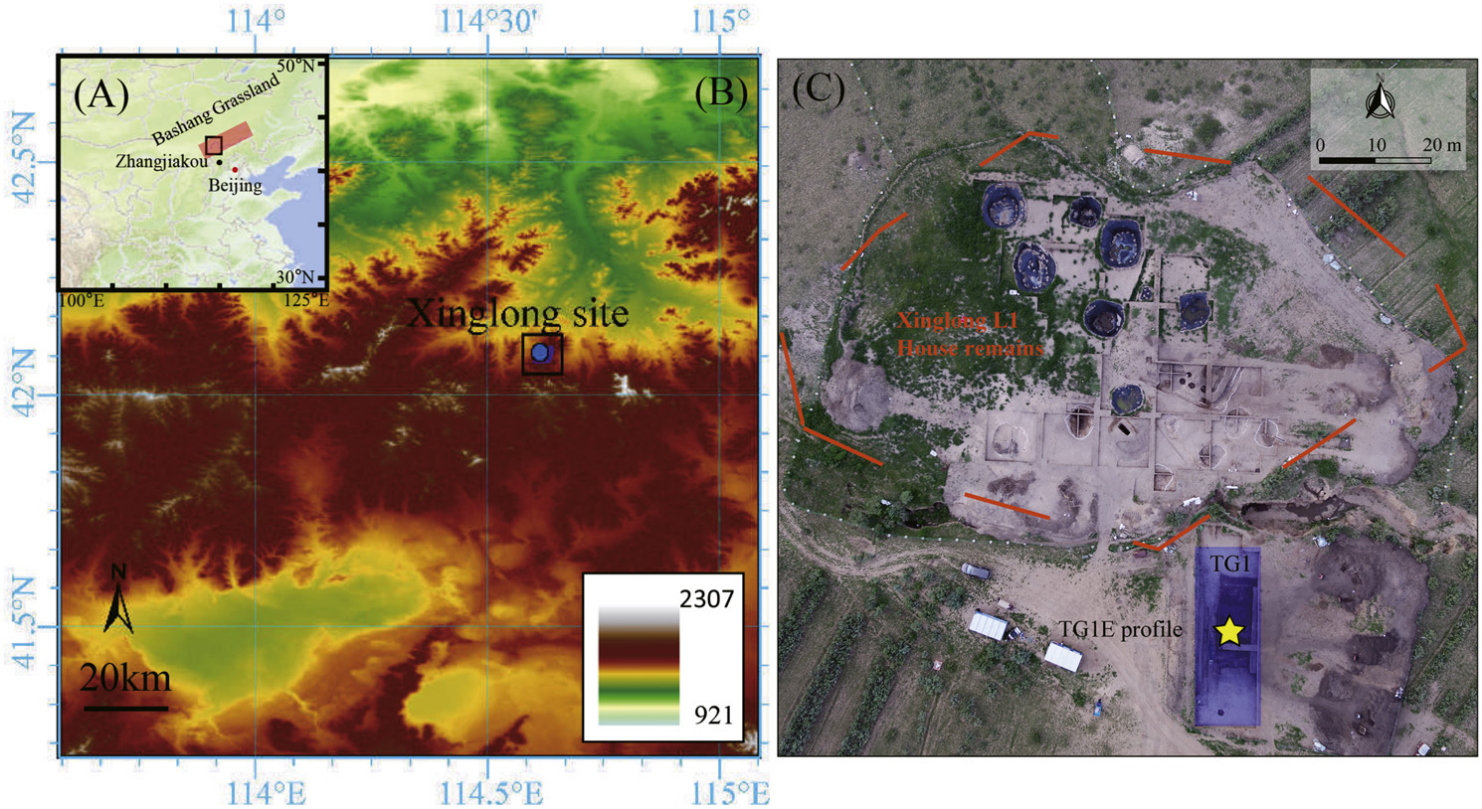
Geographical overview of the study area and sampling site (revised from Qiu et al. (2025) [1]). (A) Regional distribution of the Bashang Grassland (red rectangle) in northern China; (B) Geographical position of Xinglong site; (C) Bird’s-eye view of the 2018–2023 excavations at Xinglong site location 1 (Xinglong L1), showing the distribution of house remains (red circle) and the location of the sampled profile TG1E (yellow star).

**Fig. 2:**
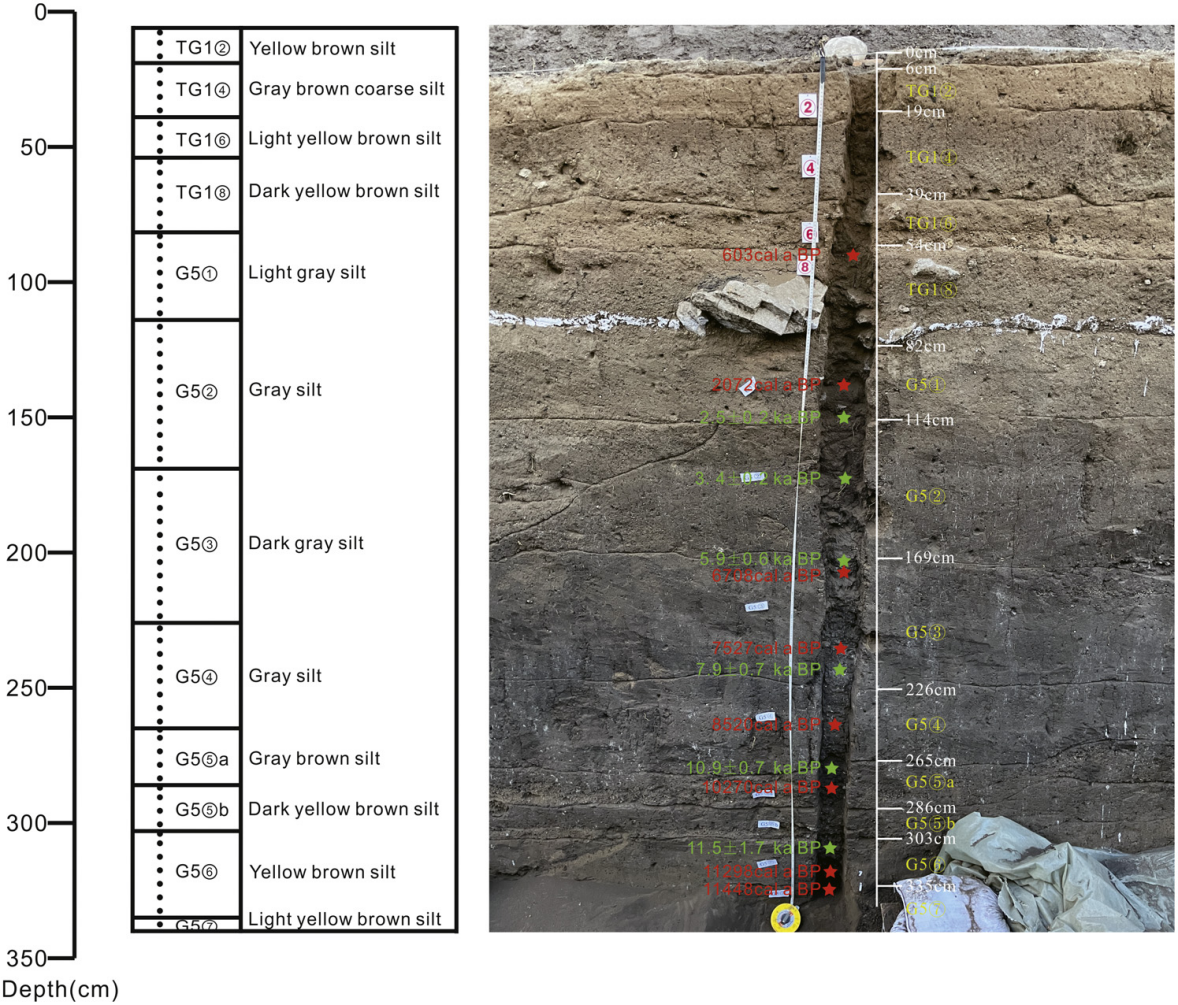
Archaeological context and dating results of the TG1E east profile at Xinglong site location 1 (revised from Qiu et al. (2025) [1]). Sampling positions (black circles) and sedimentary features are indicated in the middle column. AMS ^14^C dating (calibrated median age) locations (red stars) and OSL dating positions (green stars, data from Zhao et al. (2022)[3]) are marked in the right column.

The Xinglong site is located southeast of Xinglong Village, Zhaoyanghe Town, Kangbao County, Hebei Province, China. It lies in the southern Bashang Plateau, near the border between Hebei Province and Inner Mongolia Province (Fig. 1A and B) [1]. The site is situated on a slope west of the seasonal river Saigeda gully, with higher terrain in the northwest and lower in the southeast. Two natural gullies flank north and south boundaries and converge southeast of the Xinglong site location 1 before flowing into the Saigeda gully. Archaeological investigations revealed the natural west-east oriented gully (G5) in the southern sector of the Xinglong site location 1. Initial cross-section excavation (TG1) in 2019 uncovered microliths and animal remains from lower deposits [2]. In 2022, we extended the excavation area of TG1 two meters eastward (designated TG1E, Fig. 1C), recovering datable cultural remains including pottery shards, microliths, and animal bones from G5’s base fill (Fig. 2).

### 4.2 Sample collection

During the excavation of the Xinglong site on August 2022, we selected the thickest continuous undisturbed sedimentary sequence from the eastern profile at TG1E as the sampling area, ensuring minimal anthropogenic or natural disturbance. Stratigraphically, TG1E comprises earth fill layer (TG1E①), cultivation soil layer (TG1E②) and cultural layers from Liao-Jin period (TG1E③-⑧). For details see Guo et al. (2021) and Zhuang et al. (2025) [2^,^^4^]. G5 underlies layer TG1E⑧ and contains eight sedimentary units. While layers G5①-② represent historical deposits, layers ③-④ represent Neolithic deposits, ⑤-⑦ comprise deposits from Paleolithic- Neolithic transition. AMS ^14^C dating of G5⑥-⑦ animal bones confirms their chronology spanning the Paleolithic- Neolithic transition to the early Neolithic. AMS ^14^C dating (calibrated median age) [1] and OSL dating [3] results showed good chronological continuity, covering about 11,500 years of history since the Holocene (see Fig. 2 and Qiu et al. (2025) [1] and Zhao et al. (2022)[3] for detail). The minimal direct human disturbance makes TG1E particularly suitable for paleoenvironmental proxy analyses and archaeobotanical investigations [4^,^^5^].

After the field excavation and standard field documentation (including written records, photographic documentation, and profile drawings), we cleaned the profile surface and employed the columnar sampling technique to collect soil samples from the east wall section from bottom to the top. The sampling intervals were determined based on visible lithological layer, and standardized vertical spacing (typically 5 cm). The 340 cm column was systematically divided into 5 cm intervals for pollen, phytolith, and multi-proxy analyses. A total of 70 contiguous samples were collected within the 340 cm sediment column. Notably, certain layers were excluded from sampling due to their limited spatial distribution (primarily concentrated at the northern or southern ends of the trench) or potential contamination from surface spoil deposits.

### 4.3 Pollen analysis

Seventy pollen samples (6–340 cm depth; 127–3230 grains/g concentration) were processed at the Institute of Hydrogeology and Environmental Geology, Chinese Academy of Geological Sciences following modified HF acid protocol [6^,^^7^]. The experimental processing flow is as follows: (1) Dry the sample and grind them into powder and weigh 100 g sediment into labeled centrifuge tubes; (2) Add *Lycopodium* tracer (10,315 grains); (3) Slowly add 10 % HCl solution and heat it in a water bath for 4 h to remove carbonates; (4) Slowly add 10 % NaOH solution and heat it in a water bath for 10 min to eliminate organic matter; (5) Slowly add 70 % HF solution and stand for 12 h to dissolve silicates; (6)Sieve through 150 μm and 7 μm mesh with a fiberglass filter; (7) Centrifuge and preserve in glycerol; (8) Identify and count ≥300 grains per sample at 400 × using Olympus BX53 optical microscope.

### 4.4 Phytolith analysis

Phytolith extraction followed standard wet oxidation protocols used for extracting phytolith from sediments [8^,^^9^] at the Key Laboratory of Vertebrate Evolution and Human Origins, Institute of Vertebrate Paleontology and Paleoanthropology, Chinese Academy of Sciences. The Institute of Archaeology, National Museum of China conducted phytolith identification and statistical analysis. The detailed experimental analysis process consists of the following steps: (1) Dry the sample and grind them into powder and weigh 5 g sediment into labeled 50 ml centrifuge tubes; (2) Slowly add 30 ml of 30 % H₂O₂ solution and heat it in a water bath for 2 h to remove organic matter; (3) After the reaction slowing down, slowly add 20 ml of 10 % HCl solution for 24 h standing then dilute with distilled water and wash with water until neutral (3000 rpm, 5 min); (4) Add *Lycopodium* tracer (20,848 grains) and distilled water, dissolve completely, and centrifuge (3000 rpm, 5 min); (5) Add 5 ml ZnBr_2_ heavy liquid (2.35 g/cm^3^), mix thoroughly and centrifuge (3000 rpm, 10 min),and transfer 2 ml supernatant to a 15 ml centrifuge tube;(6) Repeat the step 5 once;(7) Wash the sample in the 15 ml centrifuge tube with distilled water and centrifuge three times (3000 rpm, 5 min), followed by ethanol-dehydrate (3000 rpm, 5 min); (8) Transfer phytolith to a 5 ml sample tube and leave it air drying; (9) Mounted on slides using Canada balsam, identify and count ≥300 grains per sample at 400 × under a Nikon Eclipse LV100N POL optical microscope.

## Limitations

Not applicable.

## Ethics Statement

We confirmed that the authors have read and follow the ethical requirements for publication in Data in Brief and confirming that the current work does not involve human subjects, animal experiments, or any data collected from social media platforms.

## CRediT Author Statement

Guanyu Wang: Data curation, Writing, Original draft. **Zhenwei Qiu:** Conceptualization, Methodology, Data curation, Writing- Reviewing and Editing. **Lina Zhuang:** Supervision. **Huiyun Rao:** Writing- Reviewing and Editing. **Wenhui Liu:** Investigation. **Zhihua Yang:** Investigation.

## Data Availability

Mendeley DataHolocene pollen and phytolith data from the Xinglong site in the Bashang Grassland, northern China (Original data)
